# Rosy Cells with a Rocky Core: Low-Grade Oncocytic Tumor with Osseous Metaplasia

**DOI:** 10.15586/jkc.v13i1.439

**Published:** 2026-01-06

**Authors:** Joseph Stenberg, Justin Tse, Emily Volpicelli

**Affiliations:** 1 Department of Pathology, University of New Mexico, Albuquerque, NM;; 2 Department of Internal Medicine, Sutter Roseville Medical Center, Roseville, CA

**Keywords:** low-grade oncocytic tumor, LOT, osseous metaplasia, renal

## Abstract

Osseous metaplasia is rarely reported in renal neoplasms and is predominantly associated with a favorable prognosis. Low-grade oncocytic tumor (LOT) represents a novel diagnostic classification of renal neoplasms that exhibit overlapping features of oncocytomas and chromophobe renal cell carcinoma. To the best of our knowledge, there are two definitive cases of low-grade oncocytic tumors with osseous metaplasia in the English literature. We present the third case of a 52-year-old female diagnosed with a low-grade oncocytic tumor exhibiting osseous metaplasia.

## Introduction

Low-grade oncocytic tumor (LOT) of the kidney was first recognized as a distinct entity in 2019 (1). Oncocytic tumors of the kidney can be challenging to diagnose due to the wide spectrum of tumor types, including oncocytoma, eosinophilic chromophobe renal cell carcinoma (RCC), and the eosinophilic variant of clear cell renal cell carcinoma (ccRCC). LOT is the newest addition to this group, and it is crucial to distinguish it from other entities given the differences in patient prognosis. Notably, all cases of LOTs reported in the literature have exhibited indolent behavior (2, 3).

The overlapping features among renal oncocytic tumors highlight the need for precise classification and diagnosis. Before LOT was officially recognized as a distinct renal tumor entity, this neoplasm might have been misdiagnosed as variants of RCC or oncocytomas. The differences in treatment options for these separate entities further emphasize the significance of this new classification.

Osseous metaplasia, characterized by the abnormal transformation of tissue stroma into bone-like tissue, is relatively rare in renal tumors. While cases of osseous metaplasia have been reported in oncocytomas, chromophobe RCC, and the eosinophilic variant of ccRCC, its occurrence in LOT has been rarely documented (4, 5). Here, we present the third reported case, identified according to the new diagnostic criteria, along with a comprehensive review of the literature.

## Case Report

A 52-year-old female with a history of myelin oligodendrocyte glycoprotein antibody-associated disease (MOGAD) presented to the emergency department with left vision changes. She had previously experienced optic neuritis flares and is currently receiving treatment with rituximab infusions, prednisone, and venlafaxine. The physical examination showed a mild relative afferent pupillary defect in the left eye, left hemianopia, and bilateral hyperreflexia. Complete blood count with differential and a comprehensive metabolic panel were both within normal limits.

**Figure 1:**
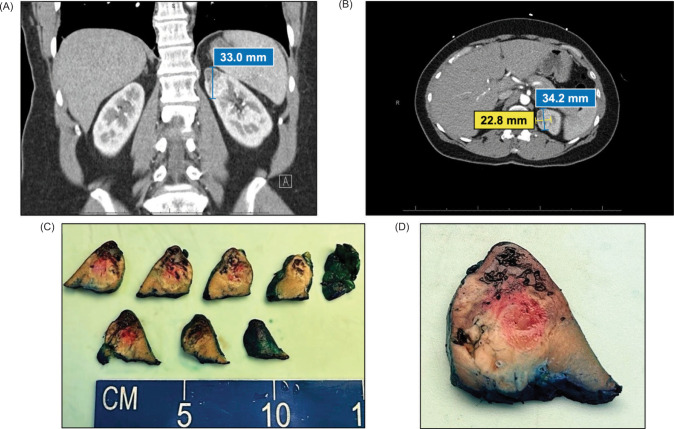
Radiographic and gross images. (A) Coronal CT of abdomen with left kidney mass measuring up to 33.0 mm; (B) Axial CT of abdomen with left kidney mass measuring 34.2 × 22.8 mm; (C) Serial sectioning of the left superior pole kidney mass; (D) Close-up image of the poorly defined mass.

A subsequent MRI of the cervical and lumbar spine revealed a partially visible lesion at the superior pole of the left kidney. Dedicated CT showed a 3.4 × 2.3 × 3.3 cm heterogeneous enhancing mass at the superior pole of the left kidney without visible calcification or necrosis (Figure 1). The right kidney appeared normal. Although bilateral inguinal lymphadenopathy was observed, it was favored to be reactive. The clinicoradiological diagnosis favored RCC, leading to a robot-assisted partial nephrectomy.

**Figure 2:**
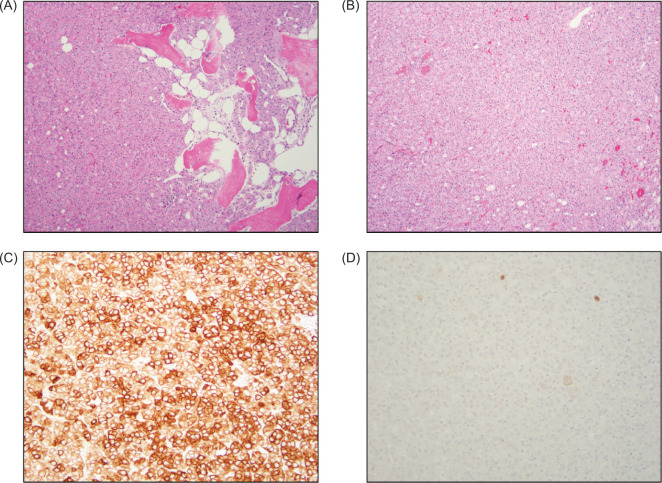
Microscopic images. **(**A) Oncocytic tumor with predominantly solid architecture and osseous metaplasia (10× objective); (B) Rosy, eosinophilic tumor cells with low-grade cytology (10× objective); (C) The tumor cells are diffusely positive for CK7 (20× objective); (D) The tumor cells are negative for CD117 (20× objective).

At gross examination, the specimen consisted of an 18 g, 4 × 4 × 3 cm partial nephrectomy. Cut surfaces revealed a 4 × 3 × 2.3 cm pale yellow, poorly defined, diffusely hemorrhagic mass closely approaching the parenchymal margin. The overlying capsule was intact (Figure 1). Histopathologic examination of the tumor revealed a rosy, oncocytic tumor with predominantly solid architecture, interspersed with edematous stromal areas and focal ossification (Figure 2). The tumor cells exhibited low-grade cytologic features. Immunohistochemistry stained positive for PAX8 and showed diffuse expression of CK7 and negative for vimentin, CAIX, and CD117/KIT (Figure 2).

Two years post-operation, the patient is alive and free of recurrence, metastatic disease, or any abnormal findings on CT scan. She continues to receive treatment for MOGAD and undergoes yearly surveillance for urologic disease.

## Discussion

Low-grade oncocytic tumor (LOT) is a renal oncocytic neoplasm that is often challenging to diagnose solely by morphological means. LOT is an indolent oncocytic tumor of the kidney characterized by solid proliferation of eosinophilic cells with low-grade nuclear features. The morphological characteristics of LOT often overlap with other renal neoplasms, including oncocytoma, eosinophilic chromophobe RCC, and the eosinophilic variant of ccRCC. The convergence of morphologic features among these entities raises diagnostic concerns, given the variability in treatment and overall prognosis. Immunohistochemistry is vital for establishing the diagnosis of LOT, as evidenced by tumor cell positivity for CK7 and PAX8 and negativity for CD117/KIT, vimentin, and CAIX (6). A summary and comparison of immunohistochemical staining patterns across oncocytic renal tumors are presented in Table 1 (6, 7).

**Table 1:** Immunohistochemistry summary of oncocytic renal tumors.

	CK7	CD117/KIT	PAX8	Vimentin	CAIX
LOT	+	-	+	-	-
Chromophobe RCC	+	+	+	-	-
ccRCC, eosinophilic variant	-	-	+	+	+
Oncocytoma	-	+	+	-	-

ccRCC, clear cell renal cell carcinoma; LOT, low-grade oncocytic tumor; RCC, renal cell carcinoma.

Calcification is a common radiological finding in both benign and malignant renal pathologies. The differential diagnosis for renal mass calcifications includes RCC, angiomyolipoma, Wilms tumor, oncocytoma, metanephric adenoma, cystic renal disease, renal abscess, schistosomiasis, echinococcal cysts, tuberculosis, xanthogranulomatous pyelonephritis, arteriovenous malformations, and hematoma (8, 9). It is documented that calcifications are present in 10–31% of RCC, and a central calcification pattern is more indicative of malignancy and a worse prognosis (10, 11). Joeckel et al. show that bone metastasis in RCC is promoted by increased calcium-sensing receptor (CaSR) expression in both the primary tumor and healthy renal parenchyma (12). Although calcium does not regulate its expression, it induces migration and proliferation of bone-metastatic RCC cells via the CaSR. This potential link may partly explain calcification and disease prognosis differences in renal tumors.

Although calcification and osseous metaplasia can appear similar on radiographic imaging, the histological features of osseous metaplasia differ and are uniquely rare in renal tumors. A MEDLINE search (using the terms osseous metaplasia, calcification, renal, kidney, and cancer) yielded only 25 results. The pathogenesis of osseous metaplasia in kidney tumors is unclear and likely multifaceted. The prevailing theory is that kidney stroma secretes a dense collagenous matrix that undergoes mineralization and organization into bone (13, 14). This suggests the metaplasia does not arise from the tumor but from the neighboring stroma. Wang et al. demonstrated that bone morphogenetic protein-2 (BMP-2) inhibits growth in human RCC cell lines ACHN and Caki-2 and induces osteogenic differentiation in pluripotent cell lines (15). Previous studies support this finding, reporting osseous metaplasia in primary tumors with evidence of BMP-2 expression in the tumor or local stroma (16–18). Interestingly, the expression of BMP-2 from either the primary tumor or adjacent stroma varies from case to case. The association of bone formation with a favorable prognosis in renal tumors is unique, and BMP-2 may provide a potential explanation. Therefore, recognition of osseous metaplasia is important, as it may affect radiologic interpretation by overestimating the risk of malignancy on preoperative imaging.

Osseous metaplasia has rarely been reported in LOT. Xie et al. have reported two cases of osseous metaplasia in LOT (4). Liu et al. reported five instances of LOT with background stroma exhibiting calcifications and/or osseous metaplasia (5); however, it is unclear how many of these cases specifically exhibited osseous metaplasia. Based on these findings, our case is the third reported LOT with osseous metaplasia. Further research is needed to establish the definitive mechanism of osseous metaplasia in renal tumors. This case underscores the need for precise histologic and immunophenotypic evaluation in renal oncocytic tumors, as imaging may not reveal calcification even in the presence of ossification.

## Conclusion

LOT is the newest addition to the spectrum of oncocytic kidney tumors, and its recognition as a distinct entity has significant implications for patient management and prognosis. Our case supports the benign nature of this tumor. Osseous metaplasia is uncommon in renal tumors and has been identified in both low-grade and high-grade kidney tumors. Careful morphologic assessment, combined with immunohistochemistry, is crucial for accurate diagnosis of this entity. Recognition of this entity, particularly when it exhibits rare histologic features such as osseous metaplasia, underscores the importance of integrating imaging and immunohistochemistry to guide management and prevent misdiagnosis.
